# Stereoregularity in Ionic Polymerization of Acenaphthylene

**DOI:** 10.6028/jres.068A.016

**Published:** 1964-04-01

**Authors:** V. M. Story, G. Canty

## Abstract

Four distinct polymers were isolated from the polymerizations of acenaphthylene initiated by boron trifluoride and *n*-butyllithium. A syndiotactic or isotactic conformation was assigned to these products on the basis of infrared and NMR evidence. The conformations and reaction details are discussed.

## 1. Introduction

A survey of the field of stereoregular polymers indicated that little has been done in deliberate control of chain conformation by means of steric hindrance of bulky groups [1].^1^,^2^ In this sense, acenaphthylene as a vinyl monomer was particularly interesting due to the extreme bulk and rigidity of the 1,8-perinaphthylene residue. Additional features were its strained five-membered ring [1a] and well defined conditions for polymerization.

The polymers of acenaphthylene have been extensively investigated. Dziewonski and his coworkers [2] were able to show ionic, thermal, and solid-state polymerization of acenaphthylene before the present-day tools for measuring high molecular weights were available. Their investigations started in 1912 and ended in 1924. Since then, other workers have examined these polymers. Jones [3] and Flowers and Miller [4], for example, have conducted extensive investigations. The only paper on the mechanism of this polymerization was by Imoto and Takemoto [5], using boron trifluoride etherate as an initiator. It should be emphasized that very little was done to measure molecular weights of these polymers [6] and no reference to possible stereoregularity was found.

A study of Fisher-Hirschfelder models of acenaphthylene polymers was made. The *cis*-isotactic polymer I, where A is in

**Figure:**
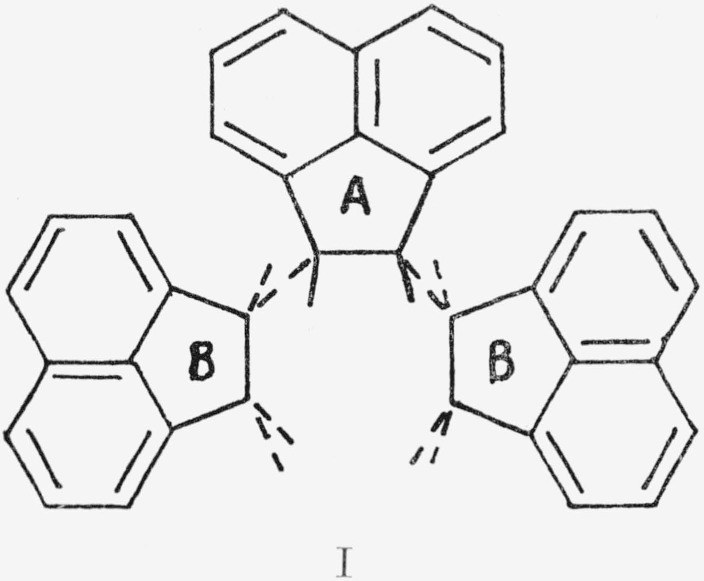

the plane of the paper and the B’s are in a parallel plane below, was self-terminating. Ring formation was favored. In fact, both a cyclic trimer and tetramer are known [2]. The *cis*-syndiotactic polymer II was impossible to construct beyond the trimer.

**Figure:**
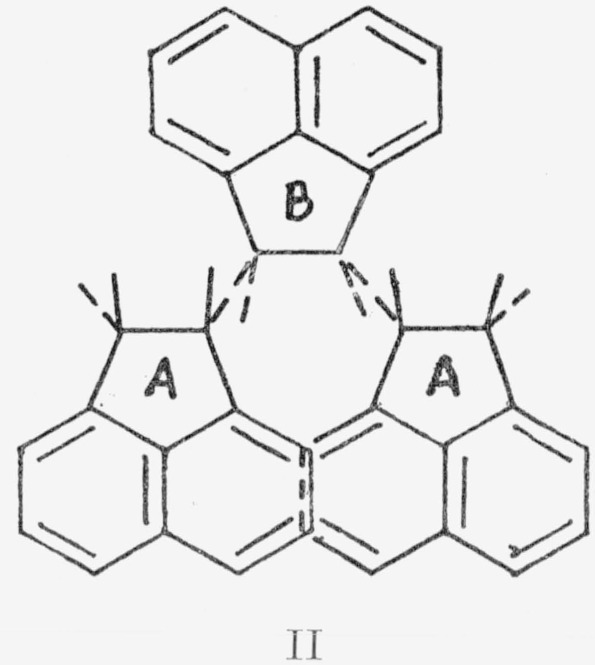

The same notation is used here as for I above. The A’s were actually twisted out of plane due to steric hindrance. *trans*-Isotactic polyacenaphthylene, III, formed a helix.^3^

**Figure:**
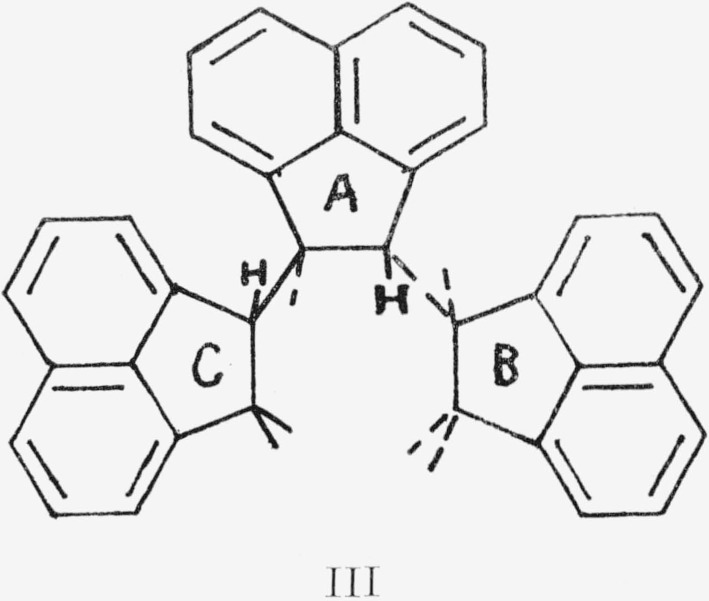

In this diagram, C is in a parallel plane above the paper, A is in it, and B below it. Similarly, the *trans*-syndiotactic polymer, IV, formed a “stair- stepped” rigid rod. The same

**Figure:**
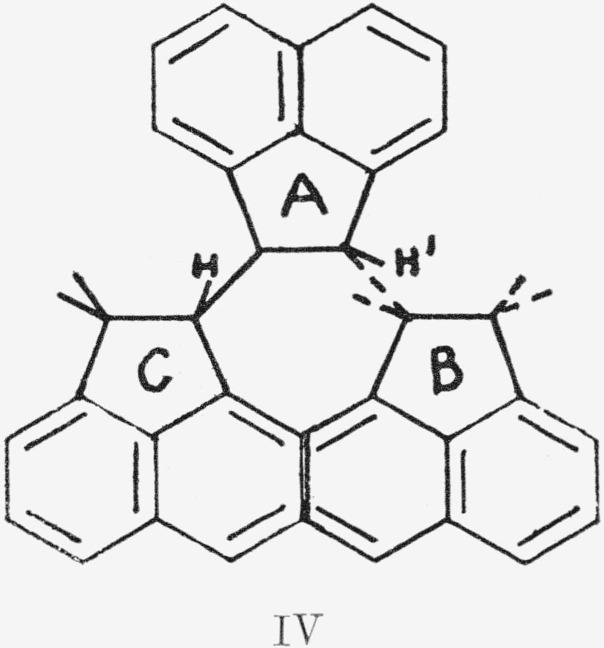

notation is used here as in III. The tertiary aliphatic protons (H) are on the inside of the helix in III and are 1,3-axial. In IV, these protons are isolated (to use the same nomenclature, they are 1,3- equatorial) from each other. H′ is adjacent to the *ortho* aromatic protons of the next residue above (C) and to the *π* cloud of the residue below (B).

The products of the polymerization of acenaphthylene with boron trifluoride and *n*-butyllithium initiators were investigated for conformations corresponding to III and IV above.

## 2. Experimental Details

### 2.1. Materials

Acenaphthylene (Aldrich Chemical Company) was sublimed twice in a vacuum apparatus. The melting point found was 92.5–93 °C. The literature value is 92–93 °C [7]. Boron trifluoride (Matheson Company, Inc.) was transferred to a glass vacuumline and distilled twice, from a 2,2,4-trimethyl- pentane slush bath (−107.4 °C), to a trap cooled by liquid nitrogen. The vapor pressure of the redistilled boron trifluoride was found to be 458 mm Hg at −107.4 °C. The literature value is 466 mm Hg [8]. Chlorobenzene and benzene (Fisher Analyzed reagents) were used without further purification. An *n*-butyllithium preparation in *n*-heptane (Foote Mineral Company, Inc.) was used as received.

### 2.2. Procedures

#### a. Polymerizations Initiated by Boron Trifluoride

The acenaphthylene was weighed into a round- bottomed flask containing a Teflon-encased magnetic stirring-bar. Chlorobenzene was added and the flask was connected to a vacuum line. The flask contents were degassed by three freeze-pump-thaw cycles. A measured volume of boron trifluoride was transferred to the frozen solution. The flask contents were permitted to warm to the required reaction temperature with stirring. The color of the reaction mixture varied from deep green to black. On completion of the reaction, the flask was vented to the air. This did not cause much color change, and presumably did not destroy all of the complex. On pouring the flask contents into boiling methanol, all color disappeared (except that of residual monomer) and a dense, white precipitate coagulated from the reaction mixture. The precipitate was filtered off, washed with cold methanol, air-dried, and dissolved in benzene, and the solution was filtered through anhydrous sodium sulfate and freeze-dried. The filtrate from the original precipitation was vacuum- evaporated. Any resulting residue was treated in the same way as for the original precipitate.

Co-initiators were added to the acenaphthylene solutions before connection to the vacuum line. In these cases, degassing was confined to one freeze-pump-thaw cycle.

A strong Tyndall effect was observed in all reaction mixtures. However, no solid separated out during or after the reactions. When sufficient water was used as a co-initiator, it was observed as ice droplets in solution or film on the sides of the flask.

#### b. Polymerization Initiated by *n*-Butyllithium

The reaction was conducted as above, except that the *n*-butyllithium solution was added through a rubber disk with a hypodermic syringe and needle. Again, the complex formed was dark green to black in color. Admission of air immediately destroyed all traces of color. After the solution had been vented to the air, it was extracted twice with 0.1 *N* HCl and washed three times with water. The organic layer was then treated as in (a) above, by precipitation in boiling methanol, etc.

### 2.3. Physical Properties

Intrinsic viscosities were determined in the standard manner at 35 °C in benzene. Infrared spectra were determined on a Perkin-Elmer 221 with a grating interchange. NMR spectra were obtained, for all polymers isolated, on a Varian A–60 instrument. Carbon tetrachloride was used as the solvent [11] and tetramethylsilane as the external standard. Ultraviolet spectra were made on a Cary Model 14 in benzene at 25 °C. X-ray photographs of powder specimens were taken with a Debye-Scherrer camera, using copper *Kα* radiation.

## 3. Results and Discussion

The results of the polymerizations of acenaphthylene, initiated by boron trifluoride and *n*-butyllithium with and without cocatalysts, are listed in table 1.

The first four reactions in table 1 duplicate the procedure of Flowers and Miller [4]. It was found that the yields from precipitation of the reaction mixture in cold methanol were not reproducible. The polymeric product was observed to redissolve on standing in the methanol-chlorobenzene mixture. An exhaustive extraction of a portion of polymer precipitated in the above manner showed an 11 percent weight loss. Since the methanol-soluble material showed the same spectra as the parent polymer in the ultraviolet region, it was concluded that oligomers were being extracted. To circumvent the coprecipitation of the oligomers, hot (boiling) methanol was used as the precipitating medium. The precipitated polymer isolated by this method will be designated “insoluble” and the product isolated from the supernatant hot methanol- chlorobenzene solution by cooling and vacuum evaporation will be designated “soluble” in the following discussion.

The residue left after isolating both the “insoluble” and “soluble” polymers was determined to be 90 to 95 percent monomer in all cases. The “insoluble” polymer isolated in the above series was designated type G.

Reactions 5 through 9, table 1, were performed using anhydrous boron trifluoride as initiator. The average total recovery, calculated on the basis of monomer weight, was 97.6 percent. The “soluble” polymer isolated in reaction 7 proved to be similar to the “insoluble” one, but of lower molecular weight.

When water or moist air was used as a cocatalyst (reactions 12 through 15, table 1), the pooled “soluble” polymer for reactions 13, 14, and 15 showed properties different from those of the “insoluble” polymer obtained in the same reactions, as described below in terms of their respective physical properties. It was designated type K.

The most striking change in products was observed when a small amount of methanol was added as co-initiator. In this case (reaction 10, table 1), the “soluble” polymer was the predominant component and proved to be a different macromolecular species, designated type M. The small amount of “insoluble” polymer recovered in this reaction was type G. Increase of the concentration of methanol co-initiator completely inhibited polymer formation, as the data for reaction 11 in table 1 indicate.

The polymerizations of acenaphthylene initiated by *n*-butyllithium gave low yields for the conditions chosen, as shown in table 1, reactions 16 and 17. The separation procedure given above for the polymerizations initiated by boron trifluoride did not give definitive results, because of low yields. The product isolated was different from all others described above and was designated type N.

The physical properties of the polymer types described above are summarized in table 2.

### 3.1. Physical Properties

#### a. Viscosities and Molecular Weights

Several molecular-weight determinations were made, to index the viscosity determinations given in table 2. An osmotic-pressure determination of one of the polymerization products ( ${\left[n\right]}_{C_{6}H_{6}}^{35{}^{\circ}}=0.036$, reaction 1, table 1) initiated by boron trifluoride gave the value 7,070 for a number-average molecular weight. The large effect of molecules of very low molecular weight on this type of measurement led us to make the same determination with a vapor-pressure osmometer. The value 2,670 was obtained by this means. This is reasonable in view of the low molecular weights and the absence of extensive purification. Taking 3,000 as an approximate number-average molecular weight, the mean degree of polymerization was about 20.

Two groups of workers have reported on the number-average molecular weight of polymers of acenaphthylene initiated by boron trifluoride. Flowers and Miller [4] stated that the moleuclar weight by osmometry was about 150,000. Imoto and Takemo to [6] reported an intrinsic viscosity of 0.040 (30°C, in benzene) and a number-average molecular weight of 125,000 using a collodion membrane. Both the above papers mention “repeated reprecipitation in methanol” from benzene solutions. However, the intrinsic viscosity value reported in the second paper indicated that extensive purification was not obtained, compared to the methods used in this study. We were not able to resolve these large discrepancies.

#### b. Infrared Spectra

**Figure 1:**
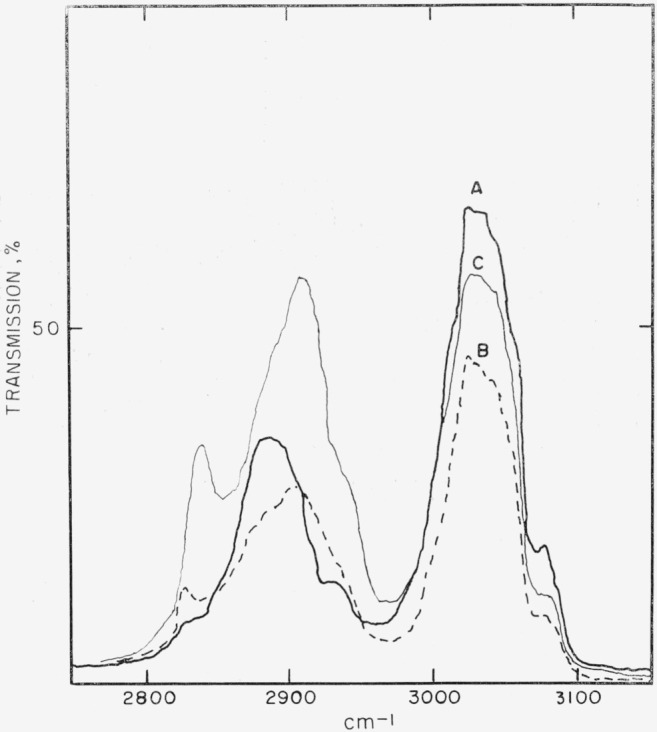
Type G polymer, spectrum *A*; type K, B; and type M, C. Ordinates, cm^−1^; abcissae in arbitrary percent transmission.

**Figure 2:**
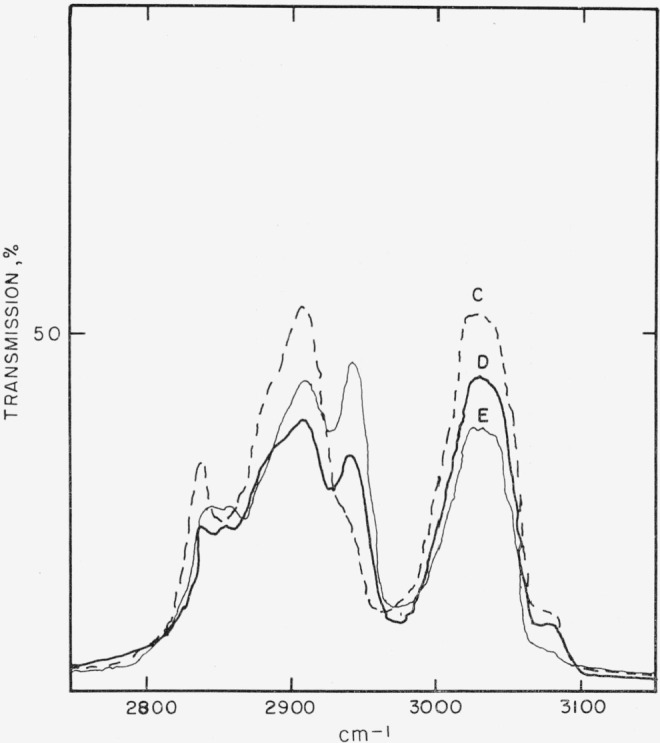
Type M polymer, spectrum *C*; type N, spectra *D* and *E*. D is the spectrum of the product isolated from reaction 16, table 1, and E of that from reaction 17. Ordinates, cm^−1^; abcissae in arbitrary percent transmission.

The saturated, tertiary carbon atoms of the backbone of poly acenaphthylene would be expected to show different C—H stretch frequencies for the *trans*-syndiotactic and *trans*-isotactic polymers. Figures 1 and 2 show the C—H stretch spectra corresponding to the four polymer types isolated in this investigation. Polymer type G, table 2, corresponds to spectrum A, figure 1; type K to spectrum B; type M to C; and type N to D and E in figure 2. The frequencies of the aliphatic C—H stretch bands of these spectra are listed in table 3, together with those of the *cis*- and *trans*-declains and acenaphthene. Although the frequencies are accurate to ± 5 cm^−1^ in this study, comparison with other spectra from the literature are probably accurate to only ± 10 cm^−1^ because of resolution differences and changes of state.

The assignment of the aliphatic C—H stretch frequency of type G polyacenaphthylene is straightforward and unambiguous, since it is almost exactly the 2890 ±10 cm^−1^ for tertiary C—H established by Fox and Martin [9]. This frequency assignment corresponds to the syndiotactic *trans* polymer (IV), as an isolated tertiary aliphatic C—H bond is unique to this structure.

Spectrum B, figure 1, contains frequencies common to both A and C. We will discuss spectrum C first, to clarify its assignments, before considering B.

A Fisher-Hirschfelder model for the *trans*-isotactic polymer, III, required 1,3-axial proton interactions similar to those of the axial bonds of cyclohexane. The closest analogs found were *cis*- and *trans*- decalin. As shown in table 3, the asymmetric C—H stretch frequency of spectrum C (type M polymer) is at 2908 cm^−1^, which is 16 cm^−1^ below that of *cis*- and *trans*-decalin at 2924 cm^−1^. By invoking a limited degree of chain flexibility for polymer configuration III, the decrease in frequency can be attributed to lower “1,3” proton-interaction energy as compared to that of the model compounds.

The symmetric C—H stretch frequency at 2840 cm^−1^ for spectrum C corresponds exactly with that of *cis*-decalin. As has been shown by nuclear magnetic resonance spectra [10], *cis*-decalin has one averaged proton-proton interaction, since the ring system is flexible. This fact suggests that the aliphatic protons in polymer M are predominantly 1,3-axial and do not interact with aromatic protons.

With the above assignments in mind, spectrum B can be rationalized as indicating the presence of either a mixture of types G and M, or a block copolymer. Spectra D and E, figure 2, corresponding to type N polymer, are clearly related to spectrum C, but have two more peaks, at 2860 and 2942 cm^−1^. The first of these can be assigned to the C—H stretch frequency of the CH_2_ group, as shown for *trans*-decalin, table 3, and the second to a combination of CH_2_ and CH_3_ frequencies. This reasoning is based on the assumption that *n*-butyl groups, derived from the *n*-butyllithium initator, were appended to one end of the polymer chain.

#### c. Nuclear Magnetic Resonance (NMR)

Acenaphthene was investigated as a reference compound under the same conditions as for the polymers. This compound has nine peaks in the aromatic proton range, from 7.07 to 7.54 parts per million of the applied magnetic field (ppm) [12, 13] and a sharp peak at 3.23 ppm corresponding to the secondary aliphatic protons. The latter value is higher than that for aliphatic protons on alkyl groups; for instance, a cyclohexane NMR spectrum gives 1.43 ppm [14]. Acenaphthene is known to have a strained ring [1a], and this fact possibly accounts for a higher screening-constant, because of reduction of the bond angle between the geminal protons.

The aromatic-proton peaks characteristic of acenaphthene were smeared into a broad peak in the same location for all of the polymer spectra. Only type G and type M polymers showed the sharp peaks characteristic of the aliphatic protons. The field shift for type G was 7.25 ppm, and for type M, 1.20 ppm.

The model studies and infrared assignments above required that the III structure have its tertiary aliphatic protons 1,3-axial to each other and on the inside of a helix. Close examination of such structures led to the conclusion that the nearest neighbor interactions were small, due to the flexibility of the backbone. The value found for M, 1.20 ppm, is in complete agreement with this reasoning, and comparison with the NMR spectra of cyclohexane (loc. cit.) supported this argument.

The structure predicted for G, IV above, showed close proximity of the tertiary protons to the *π*-electron “cloud” and to aromatic protons of adjacent aromatic residues. The chemical shift is expected to be high due to high screening of aromatic rings. The value found for G, 7.25 ppm, is in accord with structure IV.

These NMR spectra complemented the infrared assignments and indicated that the tertiary-proton environments are those predicted from the model studies of polymers G and M. Type K polymer remained indeterminate in structure, since no further information was acquired as to whether it was a mixture or a copolymer. The NMR spectrum for type N was not resolved, due, no doubt, to its higher molecular weight. (High-temperature equipment was not available to increase the resolution.) However, the infrared evidence indicated that N is a derivative of III.

#### d. Ultraviolet Spectra

It would be expected that *trans*-isotactic polyacenaphthylene would be less accessible to solvent interaction, due to its helical conformation (III), than the *trans*-syndiotactic polymer (IV) and thus show a lower extinction coefficient. The results shown in table 2 support this contention. These data are considered here to be supplementary supporting evidence for the proposed configurations.

#### e. X-ray spacings

Powder specimens of polyacenaphthylene designated type G, M, and N gave the Bragg spacings shown in table 4. The powder photographs were not sharp. The larger spacings useful for determining the chain conformation were not resolved. There were several spacings which were significantly different among the three polymer types. The 2.81 Å spacing for type G, and the 5.27 Å spacing for type N were clearly different. The 10.8 Å spacing for type M was not significantly different from the 10.3 and 10.4 Å spacings for types G and N, respectively. Thus, although differences were apparent, no definitive analysis could be made of these data to determine the crystal structures of the polymer types.

### 3.2. Mechanism

It is useful to consider the chemical nature of initiator and co-initiator complexes before inquiring into the polymerization process. Greenwood and Martin have shown that stable complexes are formed when boron trifluoride reacts with water or methanol [15]. They have also shown that the compounds hydroxyfluoroboric acid (H^+^BF_3_OH^−^), dihydroxyfluoroboric acid (H_3_O^+^BF_3_OH^−^), methoxyfluoroboric acid (H^+^BF_3_OCH_3_^−^), and dimethoxyfluoroboric acid (H_2_OCH_3_^+^BF_3_OCH_3_^−^) have definite and large conductivities in the melt [16]. These data are reported in table 5 in terms of percent ionization in the melt, together with the melting points of the fluoroboric acid compounds measured by the same authors. As solids, these compounds are reported to be partially ionized if crystallized rapidly from solution [17]. Other evidence [18] favors the ionic structures indicated in the empirical formulas. The relative amounts of ionization of the methoxyfluoroboric acids are supported by the calculations of Bender [19] from the equation of Edwards [20]. Bender’s calculations indicate that methanol is a stronger nucleophile than water. Thus, the methoxyfluoroboric acid compounds would be expected to show less ionization than the comparable hydroxyfluoroboric acids, in agreement with the results in table 5.

On the basis of infrared spectra, the greater ionization of the dihydroxyfluoroboric and dimethoxyfluoroboric acids as compared to the corresponding mono-acids (table 5) is attributed by Babushkin et al., to dimer formation, as contrasted to polymer formation for the 1:1 compounds [21]. Diehl [22] reports NMR evidence in accord with this conclusion.

Comparison of the melting points for the fluoroboric acid complexes given in table 5 with the experimental conditions for acenaphthylene polymerization given in table 1 suggest that all but one of the reactions initiated by boron trifluoride were conducted under heterogeneous conditions. As Clark has pointed out [25], traces of water are present even when extreme care is taken to produce ‘anhydrous’ boron trifluoride for vinyl polymerizations. The observed Tyndall effect must be due to the initiator complex present as a finely divided solid since the polymer is soluble in chlorobenzene. Thus, the fluoroboric acid compounds are solid under the given reaction conditions, as was excess water, but not methanol. In reaction 11, the initiator complex would be dissolved in excess methanol, in contrast to 10 where heterogeneous initiation is expected.

Several attempts were made to determine the rates of polymerization of acenaphthylene under the conditions of experiment 6, table 1. Reaction times as short as 20 sec gave the same yields. It was not feasible to extend these experiments to shorter reaction times.

The deliberate addition of water or methanol had a variable effect on the yield (reactions 10 through 15). With the exception of reaction 13, the excess of co-initiator over the initiator (on a molar basis) depressed the yield as well as changed the nature of the product. These facts suggest that the addition of co-initiator inhibits the formation of both polymers, but that the inhibition is greater for the isotactic product. The exact cause of the increase in yield of reaction 13 compared to 12 and 14 is not known.

Reactions 1 through 4 are comparable to reactions 6 through 9 in yield when corrected for oligomer concentration. These reactions were probably initiated by hydroxyfluoroboric acid while reactions 12 through 15 were initiated predominantly by dihydroxyfluoroboric acid. The degree of polymerization of reactions 12 through 15 was higher (table 2) with respect to the *insoluble* product (type G).

The above discussion is certainly oversimplified, since *both* hydroxyfluoroboric acids are present at all times, but in different proportions [18]. However, we have argued in terms of the predominant species for the sake of clarity.

Before proceeding to the case of acenaphthylene polymerization by initiation with methoxyfluoroboric acid, it is profitable to review the details of acenaphthylene polymerization initiated by hydroxyfluoroboric acid. Consideration of the pertinent data in table 1 show that temperature (footnote 3, table 1), monomer concentration, and reaction time have no discernible effect on the yield under the given reaction-conditions. The degree of polymerization of the products increases when the water concentration increases. The extinction coefficients for the polymers given in table 2 indicate that small amounts of the *trans*-isotactic polymer are present in the “soluble” product. Whether or not the isotactic polymer is or is not present as part of a block copolymer remains undecided. However, a block copolymer is unlikely at these low degrees of polymerization. The primary conclusion from the structure assignments and discussion above is that solid hydroxyfluoroboric acids give, predominantly, *trans*-syndiotactic polyacenaphthylene. It is probable, but not so well established, that the stronger dihydroxyfluoroboric acid decreases the yield, but increases the degree of polymerization.

With the above summary in mind, the heterogeneous initiation of acenaphthene polymerization by methoxyfluoroboric acid (reaction 10, table 1) has only one difference from initiations by the hydroxyfluoroboric acids. As shown in table 5, methoxyfluoroboric add is a distinctly weaker acid or, alternatively, a stronger nucleophile. The predominant polymer found is *trans*-isotactic polyacenaphthylene, with traces of the *trans*-syndiotactic polymer, in contrast to the polymerizations of acenaphthylene by hydroxyfluoroboric acids. It follows that the methoxyfluoroboric acid anion stabilizes the carbonium ion formed after donating its proton to acenaphthylene. Furthermore, the propagating polymer carbonium ion must be stabilized. The increase in nucleophilicity of the methoxyfluoroboric acid over the hydroxyfluoroboric acids is enough to favor formation of the *trans*-isotactic polymer carbonium ion over the corresponding *trans*-syndiotactic carbonium ion. The high steric hindrance of the *trans*-isotactic carbonium ion indicates that a “Sufficiently stable” complex must last longer than the comparable syndio carbonium ion, since three of the six Cartesian axes are accessible to monomer attack, compared to four in the *trans*-syndio carbonium ion.

Turning now to acenaphthylene polymerization by *n*-butyllithium, we find a clear precedent set for us. Worsfold and By water [23] have shown that previously reported cases of stereoregular polymerization of styrene [24] by *n*-butyllithium in aromatic solvents were dependent on the presence of traces of water. The experiments reported here did not rigorously exclude water in the acenaphthene polymerization with *n*-butyllithium initiation. The product obtained was *trans*-isotactic polyacenaphthylene with *n*-butyl end-groups (loc. cit.).

Although Worsfold and Bywater [23] did not believe that their experiments decided the question of “whether a colloidal surface is necessary,” their reactions were run at −30 °C where any added water is certainly frozen to ice. The generation of lithium *n*-butoxide without complete destruction of the initiator probably creates a situation analogous to that discussed above, where mixtures of solid compounds act as stereoregular initiators. The same reasoning applies in detail to reactions 15 and 16 in table 1.

The discussion above leads to the conclusion that all of the effective initiators used were heterogeneous. The nucleophilicity of the fluoroboric acid complex determined the stability of the initiating and propagating carbonium ion and, hence, the predominant stereoregular product. Traces of ice reacting with *n*-butyllithium were probably instrumental in inducing heterogeneous initiation of acenaphthylene polymerization.

## 4. Summary

The four polymers isolated from acenaphthylene polymerizations initiated by boron trifluoride and *n*-butyllithium were shown to correspond to syndiotactic or isotactic structures on the basis of infrared and NMR spectra. The syndiotactic polymer had a “rod” conformation, and the isotactic, a helix.

## Figures and Tables

**Table 1:** Ionic polymerization of acenaphthylene

Reaction number	C_12_H_8_ [*M*]^2^,^3^	Initiator,^1^ [*I*] × 10^4^	Co-initiator		Reaction time	Yield
			type	conc. [*M*] × 10^4^		

I. Initiation by boron trifluoride						

1	2.35	const.^4^	H_2_O^5^	………	45 min	92.4 6
2	2.35	const.^4^	H_2_O^5^	………	4 days	82.4 6
3	2.35	const.^4^	H_2_O^5^	………	6 days	89.0 6
4	2.35	const.^4^	H_2_0^5^	………	8 days	90.1 6
5	0.211	48.1	none 7	………	30 min	84.2
6	.211	45.0	none	………	30 min	87.0
7	.211	35.1	none	………	30 min	86.8
8	.211	11.3	none	………	30 min	74.3
9	.211	6.67	none	………	30 min	82.3
10	.211	32.7	CH_3_OH^7^	2.4	30 min	30.2^8^
11	0.201	8.85	CH_3_OH^7^	61.8	30 min	0.0
12	.201	29.1	H_2_O^7^	55.6	22 min	69.1
13	0.343	17.4	H_2_O^7^	139	30 min	82.4
14	.358	13.9	H_2_O^7^	556	30 min	49.6
15	.516	12.2	H_2_O^9^	………	30 min	87.6

II. Initiation by *n*-butyllithium^10^						

16	0.075	6.0	(7)	………	24 hr	7.0
17	.028	30	………	………	24 hr	0.7

1 [*I*] indicates the concentration (moles per liter) of ionic initiator if it all dissolved in the monomer solution.

2 The solvent for these reactions was chlorobenzene. Reactions 1–4 were done at −5 °C and the remainder at −23 °C.

3 [*M*] indicates the concentration of acenaphthylene in moles per liter.

4 A slow flow of BF_3_ was supplied to the reaction mixture.

5 Traces of water were present.

6 These reaction products were precipitated from cold methanol.

7 See experimental section for details.

8 The product soluble in boiling methanol was 30.2%, the insoluble, 2.0%.

9 Moist air was admitted to the reaction flask during the run.

10 The solvent was benezene. The reaction temperature was 21 °C. The calculated molarity of *n*-butyllithium in the final solution is reported.

**Table 2:** Polymeric products of polymerization of acenaphthylene

Polymer type^1^	Reaction (table 1)	Solubility^2^	3 ${\left[\eta \right]}_{C_{6}H_{6}}^{35^{{}^{\circ}}}$	*ϵ_A_*^4^
				
G	1–4	0	0.039±0.004	6860
	5–9	0	.034±0.001	6870
	7	+	.025 5	6570
	10	0	(6)	………
	12–15	0	.047±0.002	6890
K	12–15	+^7^	.029	6500
M	10	+	.028	5900
N	16, 17	(8)	.036, 0.100^9^	6080

1 The polymer types are as designated in the text.

2 The solubility refers to the boiling methanol chlorobenzene mixture from which the polymer is precipitated, as described in the experimental section. Reactions 1–4 were precipitated in cold methanol.

3 Two-point viscometric determinations were made. The [*η*] values were corrected for the presence of 11% oligomer for reactions 1–4 and all “soluble” products (see text). The mean and the average deviation from the mean is reported.

4 The extinction coefficient was calculated per acenaphthylene residue. The determinations were done in benzene at 2960 Å and at 25 °C. The average error was 2%.

5 The physical properties were determined on one sample, reaction 7, table 1.

6 Product was identified by its infrared spectrum. See text for details.

7 The properties of a pooled sample were determined.

8 The yield was too low to permit determination of the solubility.

9 The intrinsic viscosities of the two products were widely different, due to varying experimental conditions (table 1).

**Table 3:** Aliphatic *C—H* infrared frequencies^1^

Decalin^2^		Acenaphthylene	Polyacenaphthylene^3^			
trans-	cis-		A (type G)	B (type K)	C (type M)	D & E (type N)
						
2924	2924					2942
		2910		2908	2908	2908
			2887	2887		
		2875				
2857		2865				2860
	2841			2840	2840	2843

1 The frequencies are given in cm^−1^; the solvent was carbon tetrachloride, unless otherwise stated.

2 From American Petroleum Institute, Infrared Spectra 1086 and 1087, respectively. These were spectra taken on pure liquid samples.

3 The letter designations correspond to the spectra in figures 1 and 2. The type designations are given in the text.

**Table 4:** Bragg spacings for polyacenaphthylene^1^

Type G	Type M	Type N	Relative intensity^2^
2.07	2.07	2.06	vw
2.81	2.76	2.75	w
3.95	3.91	3.95	s
5.47	5.47	5.27	m
10.3	10.8	10.4	vs

1 The spacings are given in angstrom units, and the type designations are given in the text.

2 vw indicates very weak, w weak, m medium, s strong, and, vs very strong relative intensities.

**Table 5:** Properties of fluoroboric acid complexes^1^

Acid complex	MP	Ionization^2^
		
	°C	%
H^+^BF_3_OH^−^	5.9–6.0	10
H_3_O^+^BF_3_OH^−^	6.2	20
H^+^BF_3_OCH_3_^−^	−18.6	2
H_2_OCH_3_^+^BF_3_OCH_3_^−^	−58.1	7

1 The references are given in the text.

2 The ionization was measured in the melt at 20 °C.

## Footnotes

1 Figures in brackets indicate the literature references at the end of this paper.

2 Note added in proof: The paper by G. Natta, G. Dall’Asta, G. Mazzanti, and G. Metroni (Makromol. Chem. 69, 124, 1963) describes the polymerization of cyclobutene with Ziegler catalysts. The stereochemistry of the products are apparently identical with the case described here. Instead of *cis* or *trains*, the nomenclature *threo* or *erythro*, respectively, is used.

3 The limited rotation about the backbone carbon-carbon bonds due to steric hindrance of the 1,8-acenaphthylene residues suggests that change in the sense of the helix of III is not possible if a penultimate effect influences the course of addition. Of course, a mixture of *d* and *l* chains would be produced, unless an assymetric initiator were used.
